# 6′-β-Fluoro-Homoaristeromycin and 6′-Fluoro-Homoneplanocin A Are Potent Inhibitors of Chikungunya Virus Replication through Their Direct Effect on Viral Nonstructural Protein 1

**DOI:** 10.1128/AAC.02532-19

**Published:** 2020-03-24

**Authors:** Kristina Kovacikova, Bas M. Morren, Ali Tas, Irina C. Albulescu, Robin van Rijswijk, Dnyandev B. Jarhad, Young Sup Shin, Min Hwan Jang, Gyudong Kim, Hyuk Woo Lee, Lak Shin Jeong, Eric J. Snijder, Martijn J. van Hemert

**Affiliations:** a Department of Medical Microbiology, Leiden University Medical Center, Leiden, the Netherlands; b College of Pharmacy, Seoul National University, Seoul, South Korea; c Future Medicine Co., Ltd., Seoul, South Korea

**Keywords:** 6′-β-fluoro-homoaristeromycin, 6′-fluoro-homoneplanocin A, SAH hydrolase, alphavirus, antiviral agents, capping, chikungunya virus, nsP1

## Abstract

Alphaviruses are arthropod-borne, positive-stranded RNA viruses capable of causing severe disease with high morbidity. Chikungunya virus (CHIKV) is an alphavirus that causes a febrile illness which can progress into chronic arthralgia. The current lack of vaccines and specific treatment for CHIKV infection underscores the need to develop new therapeutic interventions. To discover new antiviral agents, we performed a compound screen in cell culture-based infection models and identified two carbocyclic adenosine analogues, 6′-β-fluoro-homoaristeromycin (FHA) and 6′-fluoro-homoneplanocin A (FHNA), that displayed potent activity against CHIKV and Semliki Forest virus (SFV) with 50% effective concentrations in the nanomolar range at nontoxic concentrations. The compounds, designed as inhibitors of the host enzyme *S*-adenosylhomocysteine (SAH) hydrolase, impeded postentry steps in CHIKV and SFV replication. Selection of FHNA-resistant mutants and reverse genetics studies demonstrated that the combination of mutations G230R and K299E in CHIKV nonstructural protein 1 (nsP1) conferred resistance to the compounds. Enzymatic assays with purified wild-type (wt) SFV nsP1 suggested that an oxidized (3′-keto) form, rather than FHNA itself, directly inhibited the MTase activity, while a mutant protein with the K231R and K299E substitutions was insensitive to the compound. Both wt nsP1 and the resistant mutant were equally sensitive to the inhibitory effect of SAH. Our combined data suggest that FHA and FHNA inhibit CHIKV and SFV replication by directly targeting the MTase activity of nsP1, rather than through an indirect effect on host SAH hydrolase. The high potency and selectivity of these novel alphavirus mRNA capping inhibitors warrant further preclinical investigation of these compounds.

## Abstract (precis)

Alphaviruses are arthropod-borne, positive-stranded RNA viruses capable of causing severe disease with high morbidity. Chikungunya virus (CHIKV) is an alphavirus that causes a febrile illness which can progress into chronic arthralgia. The current lack of vaccines and specific treatment for CHIKV infection underscores the need to develop new therapeutic interventions. To discover new antiviral agents, we performed a compound screen in cell culture-based infection models and identified two carbocyclic adenosine analogues, 6′-β-fluoro-homoaristeromycin (FHA) and 6′-fluoro-homoneplanocin A (FHNA), that displayed potent activity against CHIKV and Semliki Forest virus (SFV) with 50% effective concentrations in the nanomolar range at nontoxic concentrations.

## INTRODUCTION

Alphaviruses comprise a group of enveloped, positive-stranded (+) RNA viruses, which includes important human pathogens such as Chikungunya virus (CHIKV) and the model viruses Semliki Forest virus (SFV) and Sindbis virus (SINV). CHIKV is an arthritogenic alphavirus that is primarily transmitted by the Aedes aegypti and Aedes albopictus mosquitoes and causes a debilitating illness known as chikungunya fever. Since its isolation in the present-day Tanzania in 1952/1953 (1), sporadic CHIKV outbreaks were reported throughout the African and Asian continents (2, 3). In 2004, the virus reemerged in Kenya and then spread eastward in the form of strains belonging to the East/Central/South African lineage that were better adapted to replication in Aedes albopictus due to an A226V substitution in the E1 protein. This resulted in large outbreaks in the South West Indian ocean islands in early 2005, in India in 2005/2006, and in Asia in the following years (4, 5). A small CHIKV outbreak in the Caribbean at the end of 2013 marked its arrival in the Americas, from which over 1.5 million infections have been reported since 2014. Following its introduction in Italy (2007 and 2017) and France (2010 and 2017) on several occasions via infected travelers, CHIKV has caused limited locally transmitted outbreaks in Europe (6–9). The geographical expansion of the Aedes albopictus vector and increased human travel pose the risk that CHIKV may become endemic in new territories.

Symptomatic CHIKV infection often manifests itself by short-lived fever and recurrent joint pain, which can last for months to years (10). Despite its widespread emergence and high morbidity, antiviral medication is not available and the current treatment consists of administration of nonsteroidal anti-inflammatory drugs to alleviate pain. Over the past years, there have been efforts to develop both direct-acting and host-targeting small-molecule inhibitors into antiviral drugs to treat CHIKV infection (11). Several potent CHIKV inhibitors that interfere with the functions of individual viral nonstructural proteins or the polymerase complex have been reported, including ribavirin, 6-azauridine, mycophenolic acid, and favipiravir (T-705) (12–14). Nevertheless, the current lack of antiviral therapy for human CHIKV infections and the generally low success rate of drug development programs underscore the need to search for compounds with improved efficacy.

Alphaviruses replicate in the cytoplasm of infected cells. Following entry, the viral genome is translated into a nonstructural polyprotein, which is subsequently processed into nonstructural protein 1 (nsP1) to nsP4 (reviewed in reference 15). The 5′ end of the viral genomic and subgenomic RNAs is modified by viral enzymes to give rise to a cap-0 (m^7^GpppA) structure. This cap structure is important for the alphavirus replication cycle since it protects the viral mRNAs from degradation by host 5′-to-3′ exonucleases, enables efficient translation of viral mRNAs, and plays a role in innate immune evasion. Alphavirus capping proceeds in an unconventional reaction sequence that differs from that used by the host cell, which is confined to the nucleus. In the case of the cytoplasmic alphavirus capping reaction, a GTP molecule undergoes methylation before it is transferred onto the 5′ end of the viral RNA, making the viral mRNA capping reaction an attractive target for antiviral drug development (16).

Like cellular methylation reactions, many viral methylation reactions use *S*-adenosylmethionine (SAM) as a methyl donor to produce the cap structure at the 5′ end of viral RNAs. A by-product and feedback inhibitor of this process is *S*-adenosylhomocysteine (SAH), which is subsequently hydrolyzed by the host enzyme SAH hydrolase. Inhibition of SAH hydrolase leads to accumulation of SAH, which indirectly interferes with mRNA capping (17). SAH hydrolase was first identified as a target for antiviral compounds in 1982, and since then several inhibitors of this enzyme have been reported (17, 18). These are known to possess antitumor and antimicrobial activities and have shown potent antiviral activity against a range of negative-stranded RNA viruses, double-stranded RNA viruses, and DNA viruses; examples include pox-, paramyxo-, rhabdo-, filo-, bunya-, arena-, and reoviruses (reviewed in reference 19). Recently, we have also described SAH hydrolase inhibitors that target a broad-spectrum of (+) RNA viruses, such as some coronaviruses, Zika virus, and CHIKV (20).

Both alphavirus nsP1 and nsP2 contribute to the formation of the cap-0 structure at the 5′ end of the mRNA: nsP1 harbors the methyltransferase (GTP + SAM → m^7^GTP + SAH) and guanylyltransferase (m^7^GTP + nsP1 → m^7^GMP-nsP1 + pyrophosphate) activities, while nsP2 possesses the RNA triphosphatase activity that removes the 5′ γ-phosphate from the nascent RNA (21–23). So far, 3-aryl-[1,2,3]triazolo[4,5-*d*]pyrimidin-7(6×H)-ones were identified as selective inhibitors of CHIKV nsP1 activity, both in cell culture infection models and in *in vitro* assays with purified Venezuelan equine encephalitis virus (VEEV) nsP1 (24, 25). More recently, the CHVB series of compounds has been described, which displays a similar activity profile (R. Abdelnabi et al., unpublished data). Enzyme-based screening assays have also identified compounds that target nsP1, such as lobaric acid, a natural compound that was a hit in a CHIKV nsP1 GTP displacement assay-based screen (26). In addition, an enzyme-linked immunosorbent assay-based screening campaign of more than 1,200 compounds using VEEV nsP1 has led to the identification of at least 18 potential nsP1 inhibitors (27). Recently, a similar assay with CHIKV nsP1 has been used to screen for CHIKV nsP1 inhibitors (28). Targeting the alphavirus capping pathway thus provides a new avenue for developing specific inhibitors of this sensitive point in the alphavirus replication cycle.

Here, we report our findings from screening a library of 80 carbocyclic adenosine and selenoadenosine analogues designed to inhibit the cellular enzyme SAH hydrolase. We identified 6′-β-fluoro-homoaristeromycin (FHA) and 6′-fluoro-homoneplanocin A (FHNA) as potent CHIKV and SFV inhibitors. By selection of escape mutants and reverse engineering we identified CHIKV nsP1 as the viral target for these compounds. Biochemical assays monitoring the formation of the ^32^P-labeled m^7^GMP-nsP1 covalent intermediate indicated that nsP1 was directly inhibited by the compounds. More specifically, an oxidized form of FHNA directly inhibited the MTase activity (but not the GTase activity) of purified SFV nsP1. Taken together, these results demonstrate that the mode of action of FHA and FHNA is based on a direct inhibitory effect on nsP1 rather than inhibition of host SAH hydrolase.

## RESULTS

### FHA and FHNA inhibit alphavirus replication.

We performed a cytopathic effect (CPE) reduction assay-based screen of 80 adenosine and selenoadenosine analogues for their ability to inhibit CHIKV, SFV, and SINV replication. VeroE6 cells were incubated with compound doses in the range of 0 to 150 μM and then infected with CHIKV, SFV and SINV at a low multiplicity of infection (MOI). Following initial hit validation, we identified two compounds, FHA and FHNA, that inhibited CHIKV replication in the nanomolar range with an EC_50_ of 0.12 and 0.18 μM, respectively, without apparent cytotoxicity (50% cytotoxic concentration [CC_50_] > 250 μM). This resulted in selectivity indexes (SI) of >1,000 for both compounds. FHA and FHNA also inhibited SFV replication, although less potently, with 50% effective concentration (EC_50_) values of 3.9 and 5.2 μM, respectively. The compounds did not confer protection to infection with SINV, a more distantly related alphavirus (Table 1).

**TABLE 1:** The antiviral effect of FHA and FHNA on CHIKV, SFV and SINV replication in CPE reduction assays^*a*^

Compound	CHIKV			SFV			SINV	
	EC_50_ (μM)	CC_50_ (μM)	SI	EC_50_ (μM)	CC_50_ (μM)	SI	EC_50_ (μM)	CC_50_ (μM)
FHA	0.12 ± 0.04	>250	>1,000	3.9 ± 3.5	>250	>64		>250
FHNA	0.18 ± 0.11	>250	>1,000	5.2 ± 3.2	>250	>48		>250

a EC_50_, concentration of compound that reduces virus-induced CPE by 50%. The EC_50_ is expressed as the mean ± the standard deviation. CC_50_, concentration of compound that reduces cell viability by 50%. SI, selectivity index, calculated as CC_50_/EC_50_.

**FIG 1:**
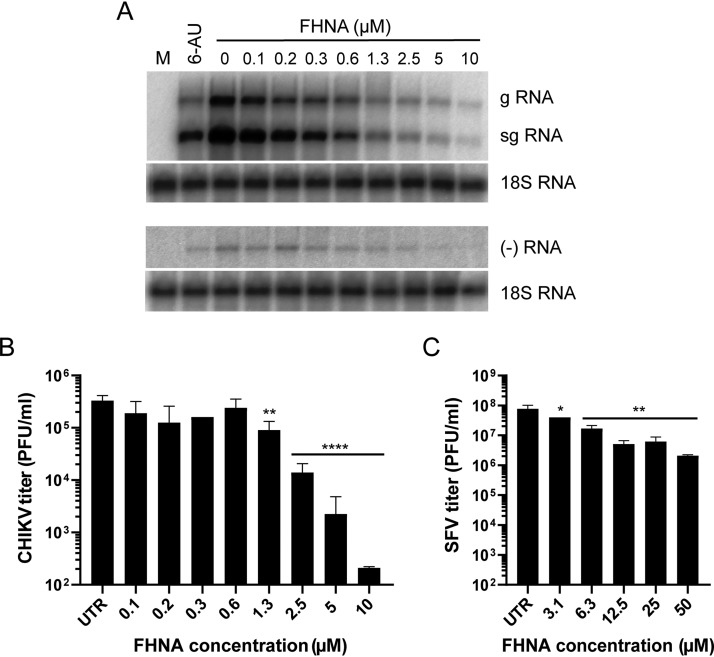
FHNA inhibits CHIKV replication in a dose-dependent manner. (A) VeroE6 cells were infected with CHIKV at an MOI of 1 and treated with 0 to 10 μM FHNA. A 20 μM dose of 6-azauridine was included as a positive control. At 8 hpi, intracellular RNA was isolated with TRIzol reagent and subjected to denaturing agarose gel electrophoresis and in-gel hybridization with ^32^P-labeled probes specific for positive- and negative-strand CHIKV RNA. 18S rRNA was used as a loading control. (B) VeroE6 cells were pretreated with 0 to 10 μM FHNA for 2 h and then infected with CHIKV at an MOI of 1. After 1 h of incubation, the inoculum was replaced with medium containing 0 to 10 μM FHNA. At 20 hpi, the supernatants were harvested for virus titration by plaque assay. (C) VeroE6 cells were pretreated with 0 to 10 μM FHNA for 2 h and then infected with SFV at an MOI of 5. After 1 h of incubation, the inoculum was replaced with medium containing 0 to 50 μM FHNA. At 8 hpi, the supernatants were harvested for virus titration by plaque assay. UTR, untreated. The data for panels B and C represent the means ± the standard deviations (SD) of two independent experiments performed in duplicate. Statistical analysis was performed using a one-way analysis of variance (ANOVA) multiple-comparison test. Statistically significant differences are indicated by asterisks (*, *P* < 0.05; **, *P* < 0.01; ***, *P* < 0.001; ****, *P* < 0.0001).

The antiviral activity of FHNA was tested in a single-cycle dose response assay by infecting VeroE6 cells with CHIKV and SFV at a high MOI, followed by treatment with 2-fold serial dilutions of the compound ranging from 0.1 to 10 μM for CHIKV and 3.1 to 50 μM for SFV. Cells were pretreated for 2 h and the compound remained present throughout the infection until samples were harvested. At 8 h postinfection (hpi), the levels of both genomic and subgenomic, as well as negative-strand, CHIKV RNA were reduced in a dose-dependent manner, while the levels of host 18S rRNA remained unchanged (Fig. 1A). FHNA also inhibited the release of CHIKV infectious progeny in a dose-dependent manner with 5 and 10 μM doses of the compound reducing viral titers by 2.5 log_10_ and 3 to 4 log_10_, respectively, compared to infected untreated cells (Fig. 1B). The inhibition of the production of SFV infectious progeny was less pronounced, nevertheless, >1 log_10_ reduction was observed already with 12.5 μM FHNA (Fig. 1C). For practical reasons, further experiments were done with FHNA because larger quantities of this compound were available at the time.

### FHNA inhibits an early step in the CHIKV and SFV replication cycle.

**FIG 2:**
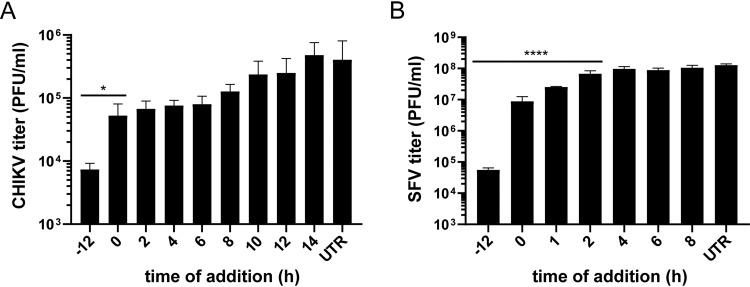
FHNA inhibits early steps in the CHIKV and SFV replication cycle. (A) VeroE6 cells were pretreated with 10 μM FHNA for 12 h, treated at the time of infection or with 2-h intervals postinfection until the time samples were harvested. The cells were infected with CHIKV at an MOI of 1, and the supernatants were harvested at 16 hpi for virus titration by plaque assay. (B) VeroE6 cells were pretreated with 50 μM FHNA for 12 h, treated at the time of infection or at 1- or 2-h intervals postinfection until the samples were harvested. The cells were infected with SFV at an MOI of 5, and the supernatants were harvested at 8 hpi for virus titration by plaque assay. UTR, untreated. The data for panels A and B represent the means ± the SD of two independent experiments performed in duplicate. Statistical analysis was performed using a one-way ANOVA multiple-comparison test. Statistically significant differences are indicated by asterisks (*, *P* < 0.05; **, *P* < 0.01; ***, *P* < 0.001; ****, *P* < 0.0001).

To investigate the inhibitory effect of FHNA on CHIKV and SFV replication in more detail, time-of-addition experiments were performed to determine the time frame during which viral infection could be inhibited. In this assay, VeroE6 cells were either pretreated with 10 μM (CHIKV) or 50 μM (SFV) of FHNA for 12 h, treated at the time of infection, or with 2-h intervals postinfection with CHIKV and SFV at a high MOI. The greatest reduction in the release of CHIKV infectious progeny occurred when the cells were pretreated, but inhibition of replication could be observed until 8 hpi (Fig. 2A). For SFV, treatment with FHNA led to a significant reduction in infectious progeny release upon pretreatment and when treatment was started before 1 hpi. When the start of treatment was postponed until 2 hpi or later, only negligible effects on SFV progeny titers were observed compared to untreated controls (Fig. 2B). In summary, maximal impairment of CHIKV and SFV replication by FHNA was observed when it was present prior to infection or during the early stages of virus replication, suggesting that uptake or metabolic conversion of the compound is slow and/or that FHNA interferes with an early step in the CHIKV and SFV replication cycle.

### Treatment with FHNA decreases CHIKV specific infectivity.

**FIG 3:**
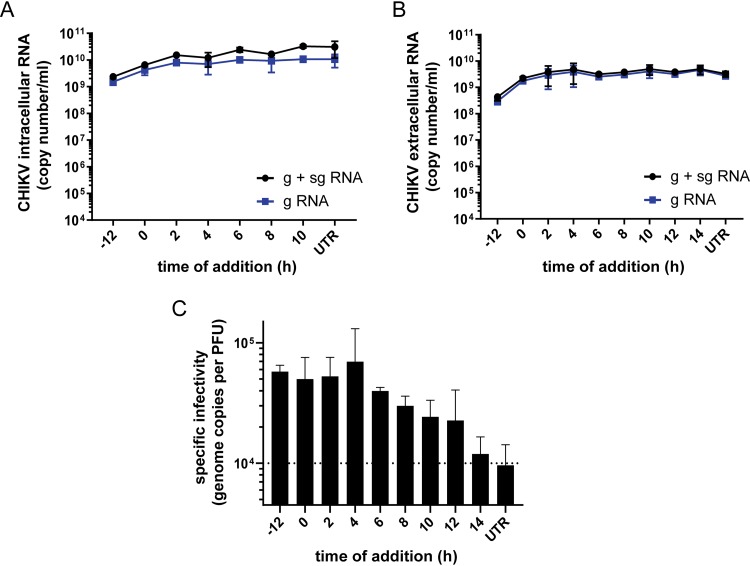
Treatment with FHNA decreases CHIKV specific infectivity. (A) VeroE6 cells were pretreated with 10 μM FHNA for 12 h, treated at the time of infection or at 2-h intervals postinfection. The cells were infected with CHIKV at an MOI of 1, the intracellular CHIKV RNA was isolated at 12 hpi, and the extracellular CHIKV RNA was isolated at 16 hpi (B). Genome copy numbers were determined by an internally controlled multiplex TaqMan qRT-PCR with probes specific for the nsP1 (gRNA) and E1 (g + sgRNA) coding regions. The data for panels A and B represent the means ± the SD of two independent experiments performed in duplicate. (C) The CHIKV specific infectivity at each treatment interval was determined by dividing the number of genome copies by the infectious virus yield (PFU/ml). UTR, untreated.

We isolated total intracellular and extracellular RNA from FHNA-treated and untreated CHIKV-infected cells at 12 and 16 hpi, respectively. CHIKV genomic and subgenomic RNA copy numbers in these samples were determined by internally controlled multiplex TaqMan quantitative reverse transcription-PCR (qRT-PCR). Treatment with 10 μM FHNA only had a limited effect on the copy numbers of intracellular genomic and subgenomic CHIKV RNA when cells were pretreated and hardly reduced copy numbers when the compound was added at the time of infection or later (Fig. 3A). Interestingly, also minimal changes in the extracellular CHIKV RNA copy numbers were observed upon FHNA treatment (Fig. 3B). This was in contrast to the clear reduction in infectious progeny titers (Fig. 2A), which led us to determine the CHIKV specific infectivity by calculating the ratio between genome copy number and infectious virus yield for the different treatment intervals. As evident from Fig. 3C, there was a decrease in specific infectivity when infected cells were pretreated or treated early in infection (−12 to 4 h). This suggests that FHNA causes a relative increase in noninfectious CHIKV particles, perhaps containing genomes unable to start an infection, e.g., because they are not capped. When 10 μM FHNA was added later during the infection (>6 h), the specific infectivity of CHIKV particles improved and gradually reached the levels of an untreated control (Fig. 3C).

### Mutations in CHIKV nsP1 confer resistance to FHNA.

**FIG 4:**
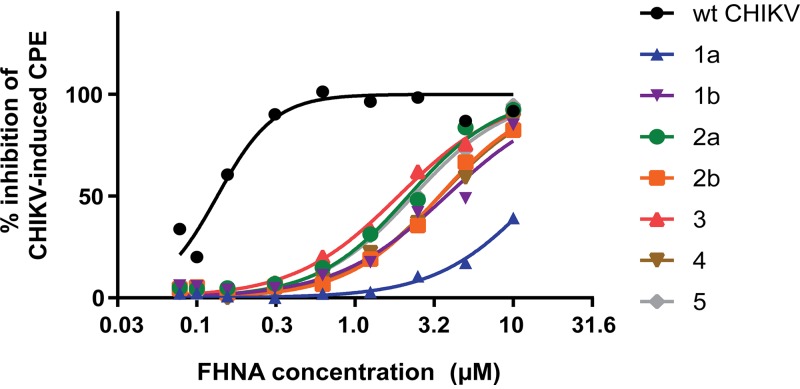
Selection of FHNA-resistant variants. Dose-response curve of the seven FHNA-resistant variants in comparison with wt CHIKV. Variants originating from the same biological replicate (progenitor) are distinguished by a letter a or b next to numbers 1 to 5.

**FIG 5:**
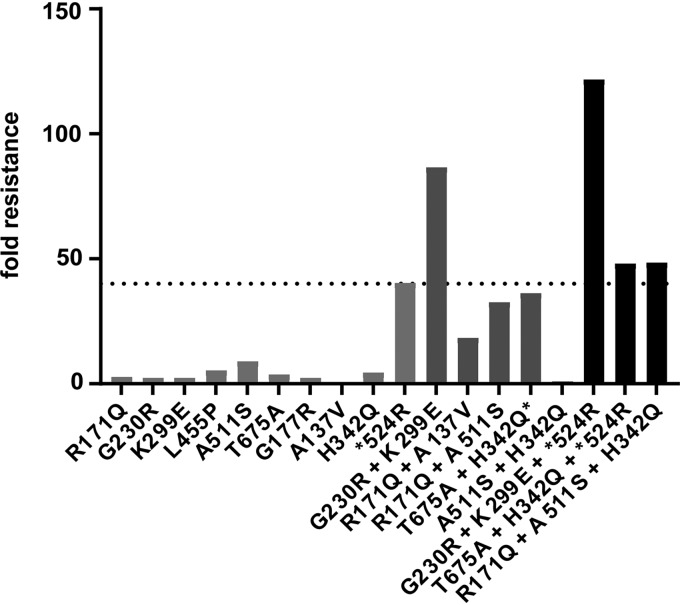
Resistance level of reverse-engineered CHIKV containing mutations selected by passaging CHIKV in the presence of FHNA in the five-step protocol. The graph shows the fold resistance to FHNA (compared to wt CHIKV) of the ten single rCHIKV mutants, five double rCHIKV mutants, and three triple rCHIKV mutants that were reverse engineered. The dashed line represents the cutoff value of 40 (level of resistance caused by the nonspecific *524R mutation) that was used to exclude mutants with a nonspecific resistance to FHNA.

In order to identify the viral target of the compound, we selected escape mutants by passaging CHIKV in the presence of FHNA. We used a previously described five-step resistance selection protocol (14), which can identify compound-resistant variants within a heterogeneous virus mixture exhibiting differing degrees of drug resistance. Mutations that are overrepresented within the resulting compound-resistant heterogeneous viral population are subsequently identified by sequencing. With this experimental setup, we selected CHIKV escape mutants using a 10 μM dose of the compound, which resulted in the isolation of seven variants (Fig. 4). The seven compound-resistant variants were substantially less sensitive to the antiviral effect of FHNA to various extents, with EC_50_ values ranging from 1.3 to 14.1 μM, compared to 0.14 μM for wild-type (wt) CHIKV (Table 2). Variant 1a showed by far the largest shift in the EC_50_ value (Fig. 4) and a >100-fold resistance compared to wt CHIKV (Table 2). To identify mutations that confer FHNA resistance, we determined the genotype of all seven compound-resistant variants by whole-genome sequencing, which revealed ten nonsilent mutations in the CHIKV open reading frame encoding the nonstructural polyprotein. We found four mutations in the nsP1-coding region, two mutations in the nsP2-coding region, and four mutations in the nsP3-coding region, some of which occurred in multiple of the seven variants, while others were unique to a particular variant (Table 3). No mutations were found in the CHIKV nsP4-coding region, the RNA-dependent RNA polymerase, suggesting that FHNA does not inhibit CHIKV by targeting viral RNA synthesis. To determine which mutation or combination of mutations was responsible for resistance, we reverse-engineered all mutations into the CHIKV-LS3 cDNA clone, either singularly or in the combinations detected in the original variants. In total, we generated 10 single recombinant CHIKV (rCHIKV) mutants, 5 double rCHIKV mutants, and 3 triple rCHIKV mutants (Fig. 5). The relative resistance levels of these rCHIKV mutants varied between 1 and 122-fold higher compared to wt CHIKV (Table 4). All single rCHIKV mutants, except rCHIKV*^524R^ (* denotes the opal stop codon UGA at the end of the nsP3 coding region) and the nonviable rCHIKV^A137V^, exhibited a 2- to 9-fold increased resistance (Table 4), indicating that a single mutation in the CHIKV genome was not sufficient to reproduce the resistance phenotypes observed for the original variants. Mutant rCHIKV*^524R^ was found to be 40-fold more resistant than wt CHIKV; however, the loss of the opal stop codon (*524R) is not specifically related to FHNA treatment, since it was also found in natural isolates and has been previously observed when CHIKV was passaged in the presence of the unrelated compound suramin (Albulescu et al., unpublished). Therefore, we chose a 40-fold increase in EC_50_ as a cutoff value (dashed line in Fig. 5), and for further analysis only considered mutants with resistance levels exceeding that of the wt virus by >40-fold (Fig. 5). Among these, variants with two mutations in nsP1, rCHIKV^G230R+K299E^ and rCHIKV^G230R+K299E+^*^524R^, were the most resistant, displaying 87- and 122-fold increases in resistance, respectively, compared to wt CHIKV (Table 4, indicated in boldface). We conclude that the combination of the G230R and K299E mutations in nsP1 is sufficient and required for phenotypic resistance, while the individual mutations hardly provide any resistance (Table 4, indicated in boldface). Importantly, the FHNA-resistant rCHIKV^G230R+K299E^ was also resistant to FHA, suggesting that both compounds have a similar mode of action. We reasoned that the higher level of resistance of rCHIKV^G230R+K299E+^*^524R^ compared to rCHIKV^G230R+K299E^ was due to the nonspecific effects of the *524R mutation.

**TABLE 2:** Phenotypes of putative FHNA-resistant variants

Variant	Mean EC_50_ (μM) ± SD	Fold resistance^*a*^
CHIKV wt	0.14 ± 0.01	1
1a	14.10 ± 0.01	100
1b	3.4 ± 0.6	24
2a	1.7 ± 0.7	12
2b	3.0 ± 0.6	21
3	1.3 ± 0.8	9
4	3.8 ± 0.3	27
5	1.8 ± 0.8	13

a The fold resistance = (EC_50_ variant/EC_50_ wt).

**TABLE 3:** FHNA-resistant variants carry mutations in CHIKV nsP1-3

Amino acid change	Protein	Variant(s)
R171Q	nsP1	4, 2a, 2b
G230R	nsP1	1a
K299E	nsP1	1a
L455P	nsP1	3
A511S	nsP2	2a, 2b
T675A	nsP2	1b
G117R	nsP3	5
A137V	nsP3	4
H342Q	nsP3	1b, 2b
*524R	nsP3	1a, 1b

**TABLE 4:** Phenotypic resistances and characteristics of all rCHIKV mutants generated by reverse engineering and compared to wt CHIKV

Recombinant virus	Mean EC_50_ (μM) ± SD	Fold resistance^*a*^
wt	0.21 ± 0.07	1
nsP1-R171Q	0.58 ± 0.37	3
**nsP1-G230R**	**0.50 ± 0.33**	**2**
**nsP1-K299E**	**0.50 ± 0.08**	**2**
nsP1-L455P	1.1 ± 1.1	5
nsP2-A511S	1.9 ± 0.6	9
nsP2-T675A	0.80 ± 0.08	4
nsP3-G177R	0.51 ± 0.02	2
nsP3-A137V^*b*^	ND^*c*^	
nsP3-H342Q	0.94 ± 0.01	5
nsP3-*524R	8.5 ± 2.3	40
**nsP1-G230R + nsP1-K299E**	**18.2 ± 4.2**	**87**
nsP1-R171Q + nsP3-A137V	3.9 ± 1.3	18
nsP1-R171Q + nsP2-A511S	6.9 ± 2.6	33
nsP2-T675A + nsP3-H342Q^*d*^	7.6 ± 0.2	36
nsP2-A511S + nsP3-H342Q	0.18 ± 0.19	1
nsP1-G230R + nsP1-K299E + nsP3-*524R	**25.6 ± 0.1**	**122**
nsP2-T675A + nsP3-H342Q + nsP3-*524R	10.1 ± 6.8	48
nsP1-R171Q + nsP2-A511S + nsP3-H342Q	10.2 ± 2.6	49

a The fold resistance = (EC_50_ recombinant mutant/EC_50_ wt). See the text for explanation of boldfacing in this table.

b Not viable.

c ND, not determined.

d The mutation H342Q reverted back to wt.

### The G230R and K299E mutations in nsP1 confer specific resistance to FHNA.

In order to assess whether the G230R and K299E mutations confer specific resistance to FHNA, we tested the sensitivity of rCHIKV^G230R+K299E^ and several other mutants to the unrelated CHIKV inhibitors mycophenolic acid (MPA) and 6-azauridine. MPA targets IMP dehydrogenase, and 6-azauridine is an inhibitor of orotidylic acid decarboxylase, leading to depletion of intracellular GTP and UTP pools, respectively (29). Both rCHIKV^G230R+K299E^ and rCHIKV^G230R+K299E+^*^524R^ exhibited minimal cross-resistance to either MPA or 6-azauridine, with a 1- to 4-fold increase in resistance compared to wt CHIKV (Table 5). Mutant rCHIKV*^524R^ displayed a 16- to 30-fold increased resistance toward both inhibitors, which are mechanistically unrelated to FHNA, once more emphasizing that this mutation causes an increase in cytopathogenicity or replication kinetics that is unrelated to specific drug resistance. Another CHIKV nsP1 mutant, rCHIKV^R171Q^, which displayed a 3-fold increased resistance to FHNA, also displayed cross-resistance to MPA (Table 5). The R171Q mutation in nsP1 was also identified independently during resistance selection for the unrelated compound suramin (Albulescu et al., unpublished) and therefore is considered nonspecific.

**TABLE 5:** Cross-resistance of FHNA-resistant and other mutants against mycophenolic acid and 6-azauridine^*a*^

rCHIKV	Mycophenolic acid		6-Azauridine	
	Mean EC_50_ (μM) ± SD	Fold resistance	EC_50_ (μM) ± SD	Fold resistance
wt	0.4 ± 0.01	1	0.3 ± 0.01	1
R171Q	4.2 ± 2.8	11	0.8 ± 0.1	3
*524R	6.3 ± 11.4	16	8.9 ± 3.4	30
G230R+K299E	1.1 ± 0.5	3	1.1 ± 0.1	4
G230R+K299E+*524R	0.5 ± 0.03	1	0.9 ± 0.1	4

a The fold resistance = (EC_50_ recombinant mutant/EC_50_ wt).

**FIG 6:**
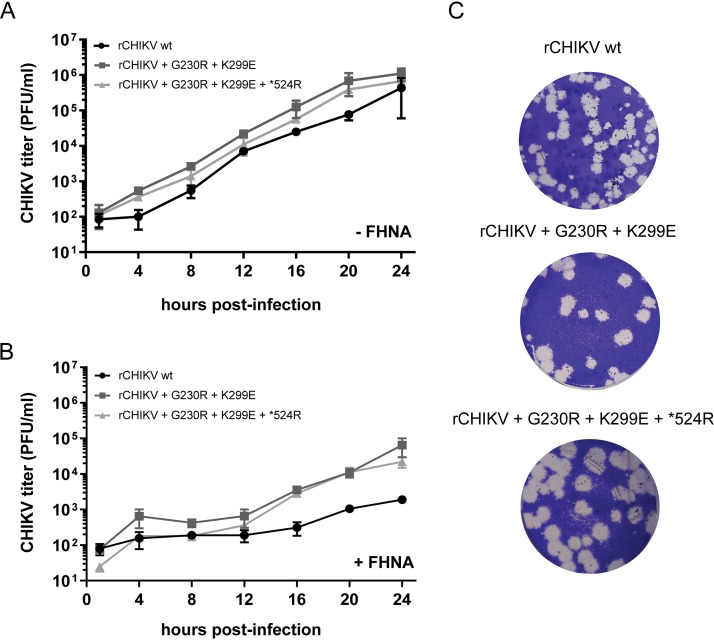
Characterization of rCHIKV with mutations in CHIKV nsp1 and opal stop codon. (A) Growth curve for selected double or triple rCHIKV mutants was performed in the absence of FHNA. VeroE6 cells were infected with CHIKV at an MOI of 1, and supernatants were harvested at 4-h intervals until 24 hpi to determine infectious progeny titers by plaque assay. (B) Growth curve for selected double or triple rCHIKV mutants was determined in the presence of 10 μM FHNA. VeroE6 cells were pretreated with 10 μM FHNA for 12 h prior to infection and infected with CHIKV at an MOI of 1. The compound remained present throughout the course of the infection. The supernatants were harvested at 4-h intervals until 24 hpi for titration by plaque assay. The data for panels A and B represent the means ± the SD of two independent experiments performed in duplicate. (C) Plaque phenotype of rCHIKV^G230R+K299^ and rCHIKV^G230R+K299E+^*^524R^ in comparison to recombinant wt CHIKV.

To assess whether the observed increased FHNA resistance is due to a nonspecific increase in replication kinetics, we performed growth curves with rCHIKV wt, rCHIKV^G230R+K299E^ and rCHIKV^G230R+K299E+^*^524R^ in the presence or absence of 10 μM FHNA. Compared to rCHIKV wt, both rCHIKV^G230R+K299E^ and rCHIKV^G230R+K299E+^*^524R^ replicated only slightly faster in the absence of compound (Fig. 6A) and produced larger plaques (Fig. 6C). In the presence of 10 μM FHNA, both mutant viruses evidently replicated better than rCHIKV wt (Fig. 6B). Nevertheless, both mutant viruses were still inhibited by the compound, since infectious progeny titers were still at least 1.5 log_10_ lower at 12 hpi and 1.2 log_10_ lower at 24 hpi in comparison to untreated controls. Taken together, the cross-resistance analysis indicated that the G230R and K299E mutations cause a FHNA-specific resistance.

### Purified SFV nsP1 with the K231R and K300E mutations is enzymatically less active *in vitro*.

**FIG 7:**
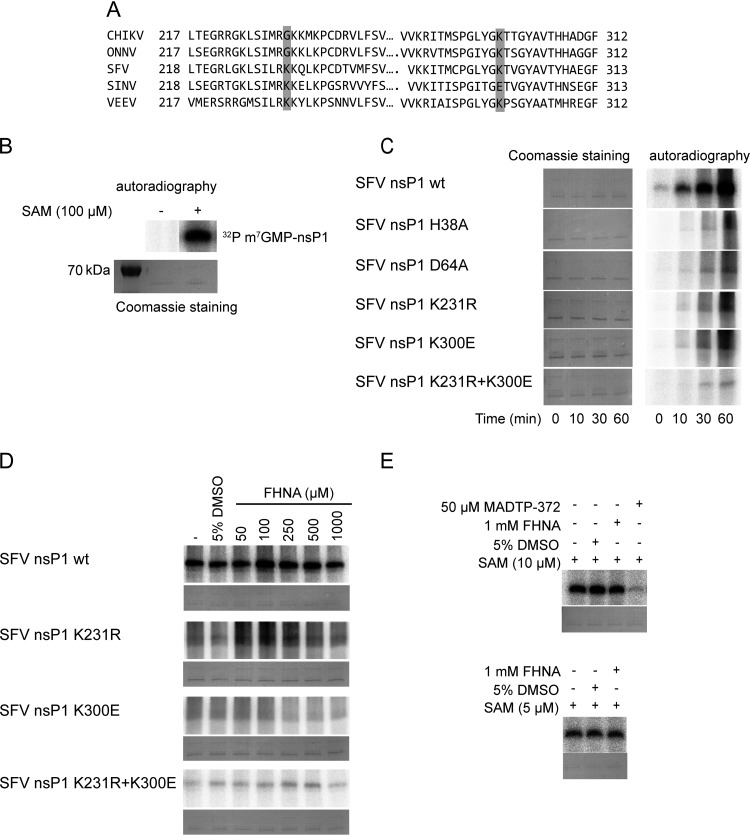
(A) Multiple sequence alignment of nsP1 of selected alphaviruses. Only specific parts of the protein near the region of interest are shown. The mutations G230R and K299E are highlighted in gray. (B) Formation of the α^32^P-m^7^GMP-nsP1 intermediate after incubation of the purified recombinant wt SFV nsP1 with α^32^P-GTP in the presence or absence of 100 μM SAM in a 30-min reaction. Coomassie blue staining with GelCode blue reagent was used to demonstrate the loading of equal protein quantities. (C) Kinetics of the α^32^P-m^7^GMP-nsP1 covalent intermediate formation of wt SFV nsP1 and mutants. The reaction mixture containing α^32^P-GTP and 100 μM SAM was stopped with 10% SDS either at *t* = 0 min (negative control) or left to proceed for *t* = 10, 30, or 60 min. (D) A dose-response assay was performed to assess the effect of FHNA on the formation of the α^32^P-m^7^GMP-nsP1 reaction intermediate by wt SFV nsP1 and mutant proteins after treatment with 50 μM to 1 mM FHNA. (E) Effect of 1 mM FHNA and 50 μM control compound MADTP-372 on the formation of the α^32^P-m^7^GMP-nsP1 covalent intermediate in the presence of 5 or 10 μM SAM. The reaction was performed at 30°C for 30 min and stopped with 10% SDS. In all cases, the α^32^P-m^7^GMP-nsP1 covalent intermediate was visualized after overnight exposure of the phosphorimager screen.

In order to study the effect of FHNA on the enzymatic activity of alphavirus nsP1 and to better understand how the CHIKV nsP1 mutations G230R and K299E may contribute to resistance, we performed *in vitro* enzymatic assays with purified nsP1. Since we were unable to obtain enzymatically active CHIKV nsP1, these assays were performed using purified wt SFV nsP1, SFV nsP1 containing either the K231R or the K300E mutation, and SFV nsP1 containing both mutations. As shown in the sequence alignment in Fig. 7A, SFV residues K231 and K300 correspond to G230 and K299 in the CHIKV nsP1 sequence. As controls, we included two previously described catalytic site mutants: SFV nsP1 ^H38A^, which is deficient in GTase activity but has retained MTase activity, and SFV nsP1 ^D64A^, which is devoid of both MTase and GTase activities (30). The enzymatic activity of these proteins was evaluated in an *in vitro* assay monitoring the formation of the covalent m^7^GMP-nsP1 complex, an important intermediate in the alphavirus capping reaction. This reaction uses α^32^P-GTP and SAM as the substrates and requires both the MTase and GTase activities of nsP1. In the presence of both substrates, a radiolabeled reaction product of ∼64 kDa (^32^P-m^7^GMP-nsP1) was indeed observed upon analysis by SDS-PAGE and autoradiography (Fig. 7B). This product was not observed when SAM was not included in the reaction, confirming the specificity of the biochemical assay (Fig. 7B). Next, we compared the activities of the various nsP1 mutants and wt nsP1, by assessing the kinetics of ^32^P-m^7^GMP-nsP1 formation (Fig. 7C, right panel) in reactions with the same amount of enzyme (Fig. 7C, left panel). The formation of ^32^P-m^7^GMP-nsP1 was clearly visible when using wt SFV nsP1, as early as 10 min after the start of the reaction, and it steadily increased over time. The active site mutants SFV nsP1^H38A^ and SFV nsP1^D64A^ were hardly active, as only trace amounts of the ^32^P-m^7^GMP-nsP1 intermediate were detected after a 30- or 60-min reaction. Interestingly, the mutant proteins with the single K231R or K300E mutation and the double mutant combining these two mutations displayed very little activity compared to wt SFV nsP1. In fact, the signal observed for these mutants was comparable to that of the active-site mutants in this assay. The K231R and K300E mutations appear to specifically affect the MTase activity, as the GTase activity of the respective proteins was unchanged in an assay (22) with m^7^GTP and SAH (data not shown).

### FHNA does not directly inhibit the enzymatic activities of SFV nsP1.

First, we investigated whether FHNA directly inhibits wt SFV nsP1 and whether SFV nsP1^K231R+K300E^ displays a differential sensitivity to the compound. To this end, *in vitro* assays were performed with wt SFV nsP1 and mutants in the presence of 50 μM to 1 mM FHNA. We did not observe any inhibitory effect of FHNA, while control compound MADTP-372 clearly inhibited the reaction (Fig. 7D and E), and we observed no difference between wt and mutant SFV nsP1 proteins in response to FHNA treatment, suggesting that the compound has no direct inhibitory effect on SFV nsP1. Since FHNA might act as a competitive inhibitor by binding to the SAM/SAH-binding site of SFV nsP1, we performed an assay with 5 or 10 μM SAM (instead of the standard 100 μM) and 1 mM compound. Our results indicated that even a 200-fold molar excess of the compound had no direct inhibitory effect on the protein’s enzymatic activity (Fig. 7E).

### The K231R and K300E mutations do not increase nsP1’s affinity for SAM or resistance to inhibition by SAH.

**FIG 8:**
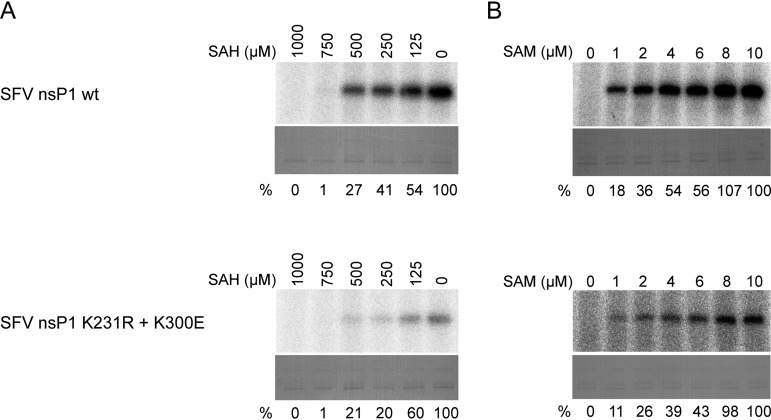
Sensitivity of wt SFV nsP1 and SFV nsP1 K231R+K300E to inhibition by SAH and dependence on SAM. (A) wt SFV nsP1 and the double mutant were incubated with [α-^32^P]GTP and 10 μM SAM in the presence of 0 to 1 mM SAH at 30°C for 30 min, and then the reaction was stopped with 10% SDS. (B) wt SFV nsP1 and double mutant were incubated with [α-^32^P]GTP and 0 to 10 μM SAM at 30°C for 30 min, and then the reaction was stopped with 10% SDS. In both panels A and B the α^32^P-m^7^GMP-nsP1 covalent intermediate was visualized after overnight exposure of the phosphorimager screen. Coomassie blue staining was used to demonstrate loading of equal protein quantities. The relative inhibition by SAH (indicated as the percentage of untreated control below the lanes in panel A) of both wt SFV nsP1 and SFV nsP1 double mutant were calculated using QuantityOne software by dividing the volume of the bands of interest by the untreated control. The relative activities at various concentrations of SAM expressed as percentages of the activity at 10 μM SAM are indicated below each lane in panel B.

Given that FHNA did not directly inhibit the formation of the covalent m^7^GMP-nsP1 intermediate, we tested whether its inhibitory effect is related to the inhibition of SAH hydrolase. Inhibition of this host cell enzyme in cell-based assays would increase intracellular SAH concentrations while decreasing SAM levels. In the *in vitro* assay, SAH clearly inhibited the enzymatic activity of wt nsP1, as the formation of the m^7^GMP-nsP1 reaction intermediate was inhibited in a dose-dependent manner by the addition of SAH (Fig. 8A). In the presence of 1 mM SAH, a 100-fold molar excess over SAM, the reaction was fully inhibited. Importantly, normalization of the activity in the presence of different concentrations of SAH to the activity of untreated control protein revealed that both wt SFV nsP1 and SFV nsP1^K231R+K300E^ were inhibited by SAH to a similar extent. Next, we assessed whether the mutations responsible for FHNA resistance lowered the affinity of SFV nsP1 for SAM, allowing the protein to be active in the presence of reduced SAM levels. There was a clear dose-dependent increase in the activity of wt SFV nsP1 when the SAM concentrations in the reaction were gradually increased from 1 to 10 μM (Fig. 8B). SFV nsP1^K231R+K300E^ behaved in a similar manner and did not appear to be more active at low SAM levels (Fig. 8B). Taken together, these results demonstrate that the K231R and K300E mutations do not increase the affinity of SFV nsP1 for SAM nor do they affect its sensitivity to inhibition by SAH.

### A metabolite of FHNA directly interferes with the enzymatic activities of SFV nsP1.

**FIG 9:**
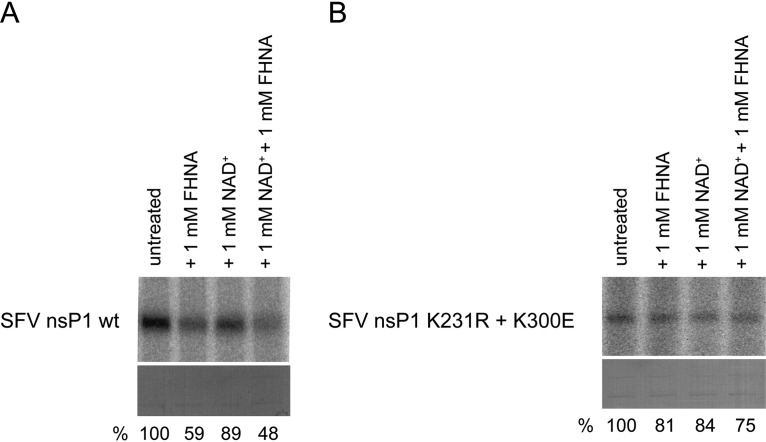
Effect of oxidized FHNA on the activity of wt and mutant SFV nsP1 under nonreducing conditions. (A) wt SFV nsP1 was incubated with [α-^32^P]GTP and 10 μM SAM and was left untreated, incubated with 1 mM FHNA, with 1 mM NAD^+^, or a combination of 1 mM FHNA and 1 mM NAD^+^ at 30°C for 30 min. The reaction was performed in the absence of DTT and was stopped with 10% SDS. (B) The reactions as described in panel A were performed with the SFV nsP1 K231R+K300E mutant. In both panels A and B the α^32^P-m^7^GMP-nsP1 covalent intermediate was visualized after a 7-day exposure of the phosphorimager screen. The relative activity (expressed as the percentage of untreated control) is indicated below the lanes in panels A and B. It was calculated using QuantityOne software by dividing the volume of the bands of interest by that of the untreated control. Coomassie blue staining was used to demonstrate equal protein loading.

Since we also identified FHNA analogues that efficiently inhibit host SAH hydrolase *in vitro* without being active against CHIKV in cell-based assays (31), we reconsidered the possibility of a direct effect of the compound on nsP1 activity. It has been previously shown that inhibition of host SAH hydrolase by halo-neplanocin A analogues is based on a mechanism that involves the oxidation of the compound to its 3′-keto form by NAD^+^ (32). Therefore, we investigated whether an oxidized (3′-keto) form of FHNA directly inhibits SFV nsP1 activity by performing enzymatic assays under nonreducing conditions and in the presence or absence of NAD^+^. Omission of dithiothreitol (DTT) from the reaction is important to allow oxidation of FHNA. Interestingly, 1 mM FHNA inhibited wt SFV nsP1 by more than 40% under nonreducing conditions, i.e., in the absence of DTT. The level of inhibition increased to more than 50% when 1 mM NAD^+^ was added into the reaction mixture containing 1 mM compound (Fig. 9A). In contrast, the SFV nsP1 mutant protein with K231R and K300E substitutions was much less sensitive to FHNA under nonreducing conditions with about 20% reduction in signal. In the presence of NAD^+^, the signal further reduced by only about 6%, suggesting that the mutant was resistant to FHNA (Fig. 9B). Therefore, we concluded that an oxidized (3′-keto) form of FHNA that can be formed under nonreducing conditions, enhanced by NAD^+^, directly inhibited SFV nsP1. We also demonstrated that the K231R and K300E mutations render the nsP1 protein less sensitive to the inhibitory effect of oxidized FHNA.

## DISCUSSION

The burden of mosquito-transmitted diseases such as chikungunya fever is expected to rise in the future due to increased global travel, climate change, and other factors. The search for antiviral drugs to treat CHIKV infections in the clinic has so far proven unsuccessful. In the present study, we identified two carbocyclic adenosine analogues, FHA and FHNA, as inhibitors of CHIKV and SFV replication. These compounds were originally designed as substrate analogue inhibitors of the host enzyme SAH hydrolase and were shown to inhibit this enzyme *in vitro* with 50% inhibitory concentration values of 0.37 and 0.91 μM, respectively (31). In CPE reduction assays, FHA and FHNA strongly inhibited CHIKV with EC_50_ values of 0.12 and 0.18 μM, respectively. They also inhibited SFV, although less potently, with EC_50_ values of 3.9 and 5.2 μM, respectively. Time-of-addition assays indicated that FHNA inhibited CHIKV and SFV rather early in their replication cycle, with, in particular, pretreatment of cells resulting in a strong reduction of CHIKV and SFV infectious progeny (Fig. 2). Pretreatment with SAH hydrolase inhibitors increases intracellular concentrations of SAH, which could indirectly interfere with SAM-dependent methylation reactions such as those involved in mRNA capping (33, 34). The inhibition of the host enzyme SAH hydrolase was shown to be responsible for inhibition of influenza virus replication, which was caused by the accumulation of intracellular SAH and reduced viral mRNA capping (35). Alternatively, pretreatment with SAH hydrolase inhibitors could be necessary to allow sufficient uptake or metabolic conversion of the compounds to their active form. We observed a reduction in CHIKV-specific infectivity upon FHNA treatment (virtually unchanged genome copy numbers, but lower infectious progeny titers in the medium), suggesting a relative increase in the production of noninfectious particles. These might contain genomes that lack a functional cap structure, since this is known to be a major determinant of alphavirus infectivity (36).

To identify the viral target of FHNA, we have selected compound-resistant CHIKV variants by repeated virus passaging in the presence of FHNA (Fig. 4). Subsequent genotyping and reverse genetics studies demonstrated that the combination of the G230R and K299E mutations in nsP1 was responsible for the specific resistance to FHA and FHNA. An additional mutation of the opal stop codon to an arginine codon (*524R) at the end of the nsP3-coding sequence further increased resistance, but this appeared to be a nonspecific effect (cell culture adaptation). In an independent study with the polyamine inhibitor difluoromethylornithine (DFMO), Mounce et al. found that the G230R mutation in CHIKV nsP1 contributes to resistance to polyamine depletion (37). This might reflect the interconnectedness of pathways linked to methionine metabolism and polyamine biosynthesis (17, 38). In contrast to our finding of the nonspecific effect of the mutation, Mounce et al. reported that the *524R mutation in combination with nsP1 mutations was important for DFMO resistance.

Alignment of alphavirus nsP1 sequences (Fig. 7A) revealed that CHIKV and O’nyong-nyong virus contain a glycine at position 230, while the corresponding position is occupied by a basic lysine residue in several other alphaviruses, including SFV. In the FHNA-resistant CHIKV variant, this residue was mutated into an arginine, another basic amino acid, which might explain why wt SFV is intrinsically less sensitive to the compound than CHIKV. At position 299/300, all aligned alphavirus nsP1 sequences except SINV contain a lysine, which was mutated to a glutamic acid in the resistant CHIKV variant. SINV already contains a glutamic acid at this position, which might explain why this virus is not sensitive to FHNA (Fig. 7A). In order to study the mode of action of FHNA in more detail and to investigate the mechanism by which the G230R and K299E mutations in CHIKV nsP1 confer drug resistance, we performed enzymatic assays with purified SFV nsP1. SFV nsP1 MTase activity (39) and GTase activity (21) were previously demonstrated using soluble fractions from Escherichia coli cells expressing nsP1. The previously described experimental conditions formed the basis for purification of enzymatically active SFV nsP1. We found that the SFV nsP1 K231R, K300E, and K231R+K300E mutants were much less active than the wt protein in an assay that measures both MTase and GTase activity using the formation of a ^32^P-labeled m^7^GMP-nsP1 as a readout. The activities of the SFV nsP1 K231R, K300E, and K231R+K300E mutants were comparable to those of the active site mutants H38A and D64A (Fig. 7C). This suggests that FHNA resistance of the K231R+K300E mutant is not simply due to higher enzymatic activity of nsP1. Furthermore, FHNA itself did not serve as a methyl donor as no reaction products were formed when SAM was substituted with FHNA (data not shown). We then explored whether the mode of action of FHNA was associated with the inhibition of the host SAH hydrolase, which leads to increased intracellular SAH levels. The enzymatic activities of purified wt SFV nsP1 and the SFV nsP1 K231R+K300E mutant were inhibited by SAH to the same extent (Fig. 8A). Moreover, both proteins exhibited a similar affinity for SAM (Fig. 8B). This is relevant for the mode of action of FHNA, since increased SAH levels in the host cell would lead to a decrease in SAM levels and interfere with SAM-dependent methylation reactions, including alphavirus nsP1-mediated mRNA capping. Our results demonstrate that FHNA resistance was not associated with increased resistance to the inhibitory effect of SAH or due to increased activity at low SAM concentrations. We then set out to investigate whether FHNA had a direct inhibitory effect on SFV nsP1, since we have also identified an FHNA analogue with a similar inhibitory activity against the host SAH hydrolase *in vitro* that is completely devoid of anti-CHIKV activity in cell-based assays (31). Previously, it was shown that halo-neplanocin A analogues inhibit SAH hydrolase via a mechanism that requires the NAD^+^ and the oxidation of the compound to a 3′-keto form (32). Therefore, we investigated the effect of FHNA on the enzymatic activity of nsP1 under nonreducing conditions and in the presence of NAD^+^. Under these conditions, wt SFV nsP1 was clearly inhibited in the presence of 1 mM FHNA and 1 mM NAD^+^ by >50% (Fig. 9A), suggesting that an oxidized form of the compound, likely the 3′ keto form, directly inhibits the MTase activity of nsP1. In contrast, the mutant protein containing the K231R and K300E mutations was much less affected by the same treatment, resulting in 25% reduction of the signal (Fig. 9B). Our results show that these mutations render the protein less sensitive to the inhibitory effect of the oxidized compound and suggest this is the basis for FHNA resistance. Of note, the overall activity of nsP1 appeared to be lower when DTT was omitted from the reaction, since the amount of ^32^P-labeled m^7^GMP-nsP1 reaction intermediate was lower compared to the standard assay that includes 5 mM DTT.

Taken together, the results of our enzymatic assays provided important insight into the molecular mechanism of FHNA-mediated inhibition of CHIKV and SFV in infected cells. Based on our findings, we argue that FHNA is predominantly a direct-acting inhibitor of alphavirus mRNA capping due to the direct effect of an oxidized form of FHNA on the SFV nsP1 MTase activity, rather than an indirect effect via the inhibition of host SAH hydrolase. We are aware that we cannot directly extrapolate the findings of the *in vitro* studies with purified SFV nsP1 to the situation in CHIKV-infected cells. Unfortunately, obtaining enzymatically active CHIKV nsP1 was not technically possible.

Alphavirus nsP1 harbors the enzymatic activities required for viral RNA capping in an N-terminal MTase-GTase domain, whereas the enzyme is also palmitoylated and associates with membranes (40, 41). In addition, it contains a membrane-binding amphipathic helix that is essential for the assembly of viral replication organelles (42). The region around position 231 of the SFV nsP1 sequence is rich in lysines and arginines, which are important for membrane binding (43). Therefore, it would also be interesting to investigate whether the FHNA-resistant nsP1 proteins, which have an additional positive charge in this region, would exhibit increased affinity for the negatively charged phospholipids found in membranes. Since it has been proposed that the membrane association of alphavirus nsP1 is required for its enzymatic activities (43, 44), the amino acid residues involved in membrane binding, such as the K231 residue in SFV, might also be important for the MTase and GTase activities. Earlier studies indicated that mutation of these basic residues (in a triple mutant of SFV nsP1 with R230A, K231A, and K232A mutations) reduced MTase activity (43). However, there are currently no alphavirus nsP1 structures available and therefore it is not yet possible to understand how our mutations map to the protein’s three-dimensional structure, how they are positioned with respect to the SAM binding pocket, and how an oxidized form of FHNA could bind to nsP1. Whether SAM and SAH use the same binding pocket or bind to different sites in the proximity of the GTP binding site also remains to be elucidated. The structures of CHIKV and SFV nsP1 might show differences that could influence their sensitivity to the compound and the effect of the mutations. The elucidation of the CHIKV nsP1 crystal structure is essential to perform compound docking studies that could explain the direct inhibitory activity of FHNA.

In summary, FHA and FHNA have been identified as potent inhibitors of CHIKV and SFV replication in cell culture. The barrier to resistance is expected to be high as two point mutations in nsP1 are required to confer resistance. Their potent antiviral activity, coupled with the fact that they target unique virus-specific enzymatic activities, warrants the further evaluation of FHA and FHNA as potential antiviral drugs to prevent or treat CHIKV infections.

## MATERIALS AND METHODS

### Cells and virus strains.

VeroE6 cells were maintained in Dulbecco modified Eagle medium (DMEM; Lonza) supplemented with 8% fetal calf serum (FCS; Bodinco), 100 IU/ml penicillin (Sigma), and 100 μg/ml streptomycin (Sigma) at 37°C in 5% CO_2_ atmosphere (DMEM–8% FCS). Infections were performed in Eagle minimal essential medium (EMEM; Lonza) with 25 mM HEPES (Lonza) supplemented with 2% FCS, 2 mM l-glutamine (Sigma), and antibiotics (EMEM–2% FCS). Baby hamster kidney (BHK-21) cells were cultured in Glasgow modified Eagle medium (Gibco) supplemented with 7.5% FCS, 10 mM HEPES, 8% tryptose phosphate broth (Gibco), and antibiotics.

CHIKV LS3 (CHIKV; GenBank accession no. KC149888) is an infectious clone-derived virus, described in Scholte et al. (45). The SFV4 strain and Sindbis virus HR small plaque strain were used in cytopathic effect reduction assays to determine the antiviral spectrum of compounds.

### Compounds.

FHA, FHNA, and their related analogues were synthesized as described elsewhere (31). The compounds were dissolved in dimethyl sulfoxide (DMSO) to obtain 20 mM stocks and were stored at 4°C until further use. MADTP-372 was dissolved as 10 mM stock in DMSO and used as described previously (24). Mycophenolic acid (MPA), 6-azauridine (6-au), *S*-adenosylhomocysteine (SAH), and guanylyl-imido-diphosphate (GIDP) were purchased from Sigma. *S*-Adenosylmethionine (SAM) and NAD (NAD^+^) were obtained from New England Biolabs. *S*-Adenosyl-[methyl-^3^H]methionine, [γ-^32^P]ATP, and [α-^32^P]GTP are products of Perkin-Elmer.

### Cytopathic effect reduction assay.

VeroE6 cells were seeded in 96-well clusters at a density of 5 × 10^3^ cells/well in 100 μl/well of DMEM–8% FCS and were allowed to adhere overnight. Next day, the medium was replaced with serial dilutions of the compounds to be tested, made in EMEM–2% FCS. Subsequently, the cells were infected with 50 μl/well of CHIKV inoculum (MOI of 0.005) or were left uninfected by adding 50 μl/well EMEM–2% FCS. Alternatively, 1 × 10^4^ VeroE6 cells/well were seeded in 80 μl/well of DMEM–8% FCS, followed by compound treatment and infection with 20 μl/well SINV or SFV inoculum (MOI of 0.025). The uninfected cells served as a control to assess potential cytotoxic/cytostatic effects of compound treatment. Each assay was performed in quadruplicate in the same plate. Cell viability was measured using the MTS/PMS [3-(4,5-dimethylthiazol-2-yl)-5-(3-carboxymethoxyphenyl)-2-(4-sulfophenyl)-2H-tetrazolium/phenazine methosulfate] method (Promega, The Netherlands) by adding 20 μl/well of MTS reagent. Depending on the virus used, this was done at 40, 76, or 96 hpi for SFV, SINV, and CHIKV, respectively. The cells were incubated for 2 h, followed by fixation with 30 μl/well of 37% formaldehyde. The optical density at 490 nm (OD_490_) was measured using an Envision plate reader (Perkin-Elmer). The 50% effective concentration (EC_50_), defined as the concentration of compound required to inhibit virus-induced cell death by 50%, and the 50% cytotoxic concentration (CC_50_), defined as the concentration of compound that reduced the OD_490_ value of uninfected cells to 50% of that of untreated control cells, were both determined using nonlinear regression with GraphPad Prism v8.0.

### Viral load reduction assay.

VeroE6 cells were seeded in 12-well clusters at a density of 1.5 × 10^5^ cells/well in 1 ml/well of DMEM–8% FCS, and were incubated overnight. The cells were pretreated with 0 to 10 μM FHNA for CHIKV and 0 to 50 μM FHNA for SFV for 2 h and infected with CHIKV at an MOI of 1 or SFV at an MOI of 5 by adding 250 μl/well of inoculum in EMEM–2% FCS with corresponding FHNA dilutions. After incubation for 1 h at 37°C on a rocker, the cells were washed three times with warm phosphate-buffered saline (PBS) and further incubated with EMEM–2% FCS in the presence of increasing concentrations of FHNA. At 8 hpi for SFV and at 20 hpi for CHIKV, 500 μl of the culture medium was harvested for viral titer determination. The cells were harvested in 500 μl of TriPure to isolate RNA for intracellular CHIKV genome copy number determination, or in 250 μl 4× Laemmli sample buffer (4× LSB) for Western blot analysis.

### Determination of viral titers.

VeroE6 cells were seeded in six-well clusters at a density of 3.5 × 10^5^ cells/well in 2 ml/well of DMEM–8% FCS, followed by overnight incubation at 37°C. Samples were 10-fold serially diluted in EMEM–2% FCS, and 500 μl/well of each dilution was used to infect confluent monolayers of VeroE6 cells for 1 h at 37°C on a rocker. The inoculum was removed and replaced with 2 ml/well of an overlay containing DMEM, 1.2% Avicel (FMC BioPolymer), 2% FCS, 50 mM HEPES, and antibiotics. After a 3-day incubation, monolayers were fixed with 3.7% formaldehyde in PBS, and plaques were visualized using crystal violet staining.

### Denaturing agarose gel electrophoresis and in-gel hybridization.

TriPure-isolated RNA samples were mixed with 1.33× formaldehyde denaturation mix (67% formamide, 23% formaldehyde, 6.7% glycerol, 10 mM morpholinepropanesulfonic acid [MOPS; pH 7.2], 6.7 mM sodium acetate, 2.7 mM EDTA, 0.07% sodium dodecyl sulfate [SDS], and 0.03% bromophenol blue). After denaturation for 15 min at 75˚C, RNAs were separated in 1.5% denaturing formaldehyde-agarose gels using the MOPS buffer system. Genomic, subgenomic, and negative-strand CHIKV RNA were detected by direct hybridization of the dried agarose gel with ^32^P-labeled strand-specific oligonucleotide probes as described by Scholte et al. (45). The probes were labeled with [γ-^32^P]ATP in a 1h reaction at 37°C containing 1 μl of T4 polynucleotide kinase (Invitrogen) and 2 μl of T4 forward reaction buffer (Invitrogen). The dried gels were first prehybridized in 5× SSPE buffer (0.9 M NaCl, 50 mM NaH_2_PO_4_, 5 mM EDTA [pH 7.4]), 5× Denhardt’s solution, 0.05% SDS, and 0.1 mg/ml Homomix I) at 55°C for 3 h, and then the ^32^P-labeled strand-specific probes were added to the buffer, followed by overnight incubation. Hybridized gels were washed twice for 15 min with 5× SSPE with 0.05% SDS. Gels were analyzed using PhosphorImager screens and a Typhoon-9410 scanner (GE Healthcare). An image of one representative experiment is shown.

### Quantitative RT-PCR.

Intracellular RNA was isolated from cells using TriPure isolation reagent (Life Technologies) according to the manufacturer’s instructions. Extracellular RNA was isolated from 150 μl of the medium of infected cells using the QIAamp viral RNA minikit (Macherey-Nagel). Both intracellular and extracellular RNA were used to determine the copy numbers of CHIKV genomic RNA (probe in nsP1-coding region, CHIKV assay 1) and total CHIKV RNA (probe in E1-coding region, CHIKV assay 2b) using internally controlled multiplex quantitative TaqMan real-time PCR. During cell lysis, the samples were spiked with a fixed amount of equine arteritis virus (EAV) to control for variations in RNA isolation or qRT-PCR efficiency. For intracellular RNA samples, PGK1 mRNA expression levels were also monitored to correct for variations in isolation or qRT-PCR efficiency. A 10-μl reaction mixture was composed of 1.25 μl of template RNA, 2.5 μl of TaqMan Fast Virus one-step master mix (Thermo Fisher Scientific), 0.5 μl CHIKV assay 1 (forward primer, AAGCTCCGCGTCCTTTACCAAG; reverse primer, CCAAATTGTCCTGGTCTTCCT; probe, 5′FAM-CCAATGTCTTCAGCCTGGACACCTT-3′ black hole quencher 1 [3′BHQ1]), 0.5 μl CHIKV assay 2b (forward primer, CTAGCTATAAAACTAAUAGAGCAGGAAATTG; reverse primer, GACTTTTCCTGCGGCAGATGC; probe, 5′ Texas Red-CGCCAGCAAGGAGGATGATGTCGGA-3′BHQ2), 0.5 μl EAV assay (forward primer, CATCTCTTGCTTTGCTCCTTAG; reverse primer, AGCCGCACCTTCACATTG; probe, 5′CY5-CGCTGTCAGAACAACATTATTGCCCAC3′-BHQ2), or PGK1 assays and 4.75 μl of nuclease-free water (Sigma). All reactions were performed in triplicate in a 384-well plate using the CFX384 Touch real-time PCR detection system and the following program: 5 min at 50°C and 20 s at 95°C, followed by 46 cycles of 5 s at 95°C and 30 s at 60°C. Data were analyzed with CFX manager 3.1 software (Bio-Rad). For absolute quantification, standard curves were generated using 10-fold serial dilutions of known quantities of *in vitro*-transcribed RNA.

### Time-of-drug-addition assay.

VeroE6 cells were seeded in 12-well clusters at a density of 1.5 × 10^5^ cells/well in 1 ml/well of DMEM/8% FCS, and were incubated overnight. The cells were pretreated with FHNA for 12 h, before they were infected with 250 μl/well CHIKV inoculum (MOI of 1) in EMEM–2% FCS containing 10 μM FHNA or SFV inoculum (MOI of 5) in EMEM–2% FCS containing 50 μM FHNA. After incubation for 1 h at 37°C on a rocker, the cells were washed three times with warm PBS to remove the unbound virus. The cells were then incubated with EMEM–2% FCS, and FHNA was added at various time points postinfection at 2-h intervals. At 16 hpi for CHIKV and 10 hpi for SFV, 500 μl of the medium was harvested for virus titration by plaque assay and RNA isolation, as described above. The intracellular RNA was isolated from the cells with the TriPure method to determine CHIKV genome copy numbers by qRT-PCR as described above.

### Selection of compound-resistant virus mutants.

A previously described five-step protocol (14) was used to select for FHNA-resistant virus variants. In the first step, VeroE6 cells were seeded in 96-well clusters at a density of 5 × 10^3^ cells/well in 100 μl/well of DMEM–8% FCS and were allowed to adhere overnight. The next day, the lowest concentration of FHNA and the highest input of CHIKV that resulted in complete block of virus-induced CPE were determined by performing antiviral assays with 500 to 1,000 PFU of CHIKV per well and 0 to 10 μM FHNA. In the second step, four 96-well clusters containing VeroE6 cells were set up per dose (5 and 10 μM) and infected with the previously determined optimal viral input (500 PFU/well). At 4 days postinfection, supernatants were collected from the five wells with the most pronounced signs of CHIKV-induced CPE. In the third step, these supernatants (potentially) containing compound-resistant variants were purified by titration in the presence of the 10 μM inhibitory dose of the compound. After a 4-day incubation, seven supernatants from wells which produced CPE at the highest viral dilution were collected from the 96-well clusters (for some original samples, two supernatants were collected). In the fourth step, reference stocks of the seven supernatants containing FHNA-resistant variants were grown in T-25 flasks, which were harvested after 3 to 4 days, when full CPE was observed. After determination of the viral titers by plaque assay, the reference stocks were used for resistance phenotyping as described below. At the same time, the resistance genotype was determined by full-genome Sanger sequencing as described below. The virus variants obtained after the five selection rounds are referred to as “P5 variants.”

### Reverse genetics.

Mutations were reverse engineered into the CHIKV LS3 full-length cDNA clone using the QuikChange site-directed mutagenesis kit (Agilent) according to the manufacturer’s instructions (primer sequences are available upon request). The constructs were verified by sequencing using 18 primers covering the whole CHIKV genome sequence (Leiden Genome Technology Center).

### Sequencing of virus genomes.

Four overlapping PCR amplicons were generated from CHIKV RNA via cDNA synthesis using RevertAid H Minus reverse transcriptase (Thermo Fisher Scientific), RiboLock RNase inhibitor (Thermo Fisher Scientific), 5× reaction buffer, 10 mM deoxynucleoside triphosphate mix, and primers (sequences available upon request). In the second step, combinations of primers (sequences available upon request) were used to generate five PCR products with the following program: 5 min at 95°C, followed by 30 cycles of 30 s at 95°C, 30 s at 55°C, and 3 min at 72°C and terminated by 10 min at 72°C. Amplicons were gel purified and sequenced using 18 primers (sequences available upon request). The nucleotide sequences were assembled in Geneious software 9.1.5, and the complete genomes of the resistant variants were compared to the CHIKV-LS3 genome.

### Resistance and cross-resistance phenotypic assay.

Essentially the same protocol was used as described above for the CPE reduction assays, with the modification that 10-fold-higher MOIs were used (MOI of 0.05, meaning 500 PFU/well). MPA and 6-au were included for cross-resistance evaluation of reverse-engineered viruses.

### Growth kinetics determination.

VeroE6 cells were seeded in 24-well clusters at a density of 7.5 × 10^4^ cells/well in 0.5 ml/well of DMEM–8% FCS and incubated overnight. Half of the clusters were pretreated with 10 μM FHNA for 12 h, and half were left untreated. The infections with recombinant CHIKV were performed with 250 μl/well inoculum (MOI of 1) in EMEM–2% FCS in the presence or absence of 10 μM FHNA. After incubation for 1 h at 37°C on a rocker, the cells were washed three times with warm PBS and further incubated with 500 μl/well EMEM–2% FCS with or without 10 μM FHNA. At 1, 4, 8, 12, 16, 20, and 24 hpi, the supernatants were harvested for determination of the viral titers by plaque assay (see above).

### Cloning, expression, and purification of wild-type and mutant SFV nsP1.

The DNA sequence encoding SFV nsP1 (amino acids 1 to 537 of the SFV replicase polyprotein) carrying a C-terminal hexahistidine (6×His) tag was cloned between the NcoI and XmaI restriction sites of the pET34 vector, downstream of the T7 RNA polymerase promoter. To generate mutants, site-directed mutagenesis was performed using a QuikChange site-directed mutagenesis kit according to the manufacturer’s instructions (primers are available on request). All mutations were confirmed by Sanger sequencing. Proteins were expressed in E. coli Rosetta cells (Novagen) by induction with 0.5 mM isopropyl-β-d-thiogalactopyranoside (IPTG) in yeast extract-tryptone (2×YT) medium (16 g of Bacto tryptone, 10 g of yeast extract, and 5 g of NaCl in 1 liter), after theOD_600_ of the culture grown at 37°C had reached 0.6 to 0.7. After 16 h of incubation at 19°C, bacterial cells were harvested by centrifugation at 5,000 rpm for 15 min at 4°C. The cell pellets were then resuspended in 10 ml of resuspension buffer (20 mM HEPES [pH 7.5], 300 mM NaCl, 5% glycerol) supplemented with one tablet of EDTA-free protease inhibitor cocktail (Roche), 10 mg of freshly dissolved lysozyme (Merck), 300 U of DNase I, and 3 mM MgCl_2_. After incubation on a rotor for 30 min at 4°C, the cells were disrupted by sonication. Soluble protein fractions containing wt or mutant SFV nsP1 were obtained by centrifugation of the lysate at 10,000 × *g* for 30 min at 4°C. This soluble fraction was used for batch protein purification under native conditions with 500 μl of Talon beads (TaKaRa). Talon beads were equilibrated in 10 ml of resuspension buffer. Next, 10 ml of soluble fraction was incubated with the beads on a horizontal shaker for 1 h at 4°C. The beads were washed three times with 10 ml of wash buffer (20 mM HEPES [pH 7.5], 300 mM NaCl, 5% glycerol, 30 mM imidazole) by centrifugation for 5 min at 2,000 × *g* and 4°C. The protein was eluted from the beads in 1 ml of elution buffer (20 mM HEPES [pH 7.5], 300 mM NaCl, 5% glycerol, 300 mM imidazole) by first incubating the beads in elution buffer at room temperature for 15 min and then pelleting the beads at 2,000 × *g* for 5 min at 4°C. This step was repeated once more to remove any residual protein from the beads. The eluted protein was concentrated, and the buffer was exchanged using Amicon Ultra-15 ultrafiltration units (Merck) and storage buffer containing 25 mM HEPES (pH 7.5) and 100 mM NaCl. Proteins were stored at −80°C. The concentration of purified proteins was determined by Bradford protein assay (Bio-Rad), and their purity was assessed by SDS-PAGE and Coomassie blue staining with GelCode blue stain reagent (Thermo Fisher).

### nsP1 enzymatic activity assay (monitoring formation of m^7^GMP-nsP1 reaction intermediate).

The covalent m^7^GMP-nsP1 intermediate formation assay protocol was adapted from (22, 30). The activity of SFV nsP1 was measured in 30 μl of mixture containing 25 mM HEPES (pH 7.5), 5 mM DTT, 10 mM KCl, 2 mM MgCl_2_, 100 μM SAM, 0.75 mCi of [α^32^P]GTP (3000 Ci/mmol), and 500 nM wt or mutant SFV nsP1. The reaction mixture was incubated at 30°C for 30 min, and the reaction was stopped by adding 3 μl of 10% SDS. Alternatively, assays were performed under nonreducing conditions by omitting DTT from the reaction as described above, and in some samples 1 mM NAD^+^ was added. Next, reactions were mixed with 4× LSB, and then 10-μl samples were resolved on a 10% SDS-PAGE gel. The gel was stained using the Coomassie method with GelCode blue stain reagent to check for equal protein loading. Subsequently, the gel was dried, and a phosphorimager screen was placed on top. After overnight or a 7-day (reactions without DTT) exposure, the ^32^P-labeled covalent m^7^GMP-nsP1 intermediate products were visualized with a Typhoon imager (Amersham).
